# Avoiding the self-nucleation interference: a pH-regulated gold *in situ* growth strategy to enable ultrasensitive immunochromatographic diagnostics

**DOI:** 10.7150/thno.70092

**Published:** 2022-03-14

**Authors:** Hong Duan, Tongtong Ma, Xiaolin Huang, Bao Gao, Lingyan Zheng, Xirui Chen, Yonghua Xiong, Xiaoyuan Chen

**Affiliations:** 1 State Key Laboratory of Food Science and Technology, School of Food Science and Technology, Nanchang University, Nanchang 330047, P. R. China; 2 Beijing Engineering and Technology Research Center of Food Additives, Beijing Technology & Business University, Beijing 100048, P. R. China; 3 Departments of Diagnostic Radiology, Surgery, Chemical and Biomolecular Engineering, and Biomedical Engineering, Yong Loo Lin School of Medicine and Faculty of Engineering, National University of Singapore, Singapore, 119074, Singapore; 4 Clinical Imaging Research Centre, Centre for Translational Medicine, Yong Loo Lin School of Medicine, National University of Singapore, Singapore 117599, Singapore; 5 Nanomedicine Translational Research Program, NUS Center for Nanomedicine, Yong Loo Lin School of Medicine, National University of Singapore, Singapore 117597, Singapore

**Keywords:** Immunochromatographic assay, Gold *in situ* growth, Hydroxylamine, Signal amplification

## Abstract

**Background:** Gold nanoparticle-based immunochromatographic assay (AuNP-ICA) has insufficient sensitivity due to its inherent colorimetric signal intensity and low capture efficiency of AuNPs. The metal *in situ* growth is a common strategy to enhance the sensitivity of AuNP-ICA due to its superior signal amplification potential and simple operation. However, the detection distortion caused by metal self-nucleation during the growth process can seriously affect the accuracy and reproducibility of the strips.

**Methods:** We present a pH-regulated gold *in situ* growth (GISG) strategy to amplify the colorimetric signal and demonstrate its application in improving the performance of traditional AuNP-ICA. The controllable growth signal amplification is achieved by lowering the pH of the growth solution to weaken the reducibility of hydroxylamine (HA), thus urging the crystallization and growth of Au^3+^ on the AuNP surface instead of free reduction and self-nucleation. In addition, the mechanism of pH regulation on HA reducibility is elucidated by introducing an electron-donating or electron-withdrawing group to affect the electron density of hydroxyl group.

**Results:** The proposed GISG strategy shows improved sensitivity, low background, robust operation, and good reproducibility. The LOD values of the designed GISG-amplified AuNP-ICA are as low as 0.0198 ng mL^-1^ for hepatitis B surface antigen and 0.0125 ng mL^-1^ for HIV-1 capsid p24 antigen, which are lower by about 500- and 70-fold, respectively, than those of the unamplified AuNP-ICA.

**Conclusions:** This method is extended to enable ultrasensitive and rapid diagnosis of viral infections, and has potential as a general signal amplification platform to redefine immunochromatographic diagnostics.

## Introduction

Gold nanoparticle-based immunochromatographic assay (AuNP-ICA) is one of the most popular point-of-care (POC) diagnostic devices, and has been widely used in various areas ranging from clinical diagnosis to food safety and environmental monitoring due to its simplicity, portability, low cost, and user-friendly features 1-8. However, traditional AuNP-ICA has the sensitivity in the range of ng mL^-1^ to μg mL^-1^ because of the relatively weak colorimetric signal brightness of 20-40 nm AuNPs and low immunoreaction efficiency (≤ 5%) of AuNP probes on the test (T) line 9-12.The suboptimal sensitivity of conventional AuNP-ICA is approximately 1-2 orders of magnitude lower than that of laboratory-based immunoassays, such as enzyme-linked immunosorbent assay and chemiluminescent immunoassay 13-15, and is far below the concentration ranges of some clinically relevant analytes especially in the early and recurrent stages of the disease 16-18. Therefore, the detection limit (LOD) of traditional AuNP-ICA needs to be lowered substantially to achieve comparable sensitivities to laboratory test methods, thus facilitating its further application in certa*in situ*ations requiring high sensitivity.

Several available strategies, including using AuNPs with high colorimetric signal brightness as alternatives 10, 11, 19-21, enhancing the capture efficiency of AuNP probes at the T zone 22-25, and conducting the in-situ signal amplification of AuNPs by nanoparticle aggregation 26-28, enzymatic deposition 29, 30, and metal growth 31-34, have been presented to enhance the detection sensitivity of traditional AuNP-ICA. Among these strategies, the metal *in situ* growth (MISG) has obtained the most widespread use due to its superior signal amplification potential and simple operation 12, 35, 36. The MISG strategy generally depends on the deposition of metal shell onto the surface of AuNPs to enlarge the size of AuNPs and amplify the colorimetric signal on the detection area. An ideal MISG strategy should ensure the crystallization and growth of metal ions on the surface of AuNP probes rather than self-nucleation, which is the biggest challenge encountered by traditional MISG techniques. The unwanted self-nucleation of metal ions can cause high background on the test strip, thereby resulting in low reproducibility and false-positive results. In a typical MISG process, metal ions, such as Au^3+^, Ag^+^, and Cu^2+^, are reduced to metal shells and then deposited on the surface of AuNPs in the presence of reducing agents 37. The kinetics of nucleation and crystal growth of metal ions is determined by the reducing power of reductants. Currently, the reductants involved in MISG include hydroxylamine (HA) 38, hydroquinone 39, 40, and ascorbic acid 41. HA is the most commonly used reducing agent for the gold *in situ* growth (GISG) strategy and has been reported to show 2-3 orders of magnitude improvement in the detection sensitivity compared with unamplified AuNP-ICA 31, 42. Nevertheless, the traditional GISG strategy by using HA as a reductant suffers the huge risk of Au self-nucleation on the test strip owing to the superfluous reducibility of HA at high pH of 7.0 over the pKa of HA (5.80-5.90) 43. Although this dilemma can be effectively alleviated by strictly controlling HA concentration and reducing time, this delicate operation remarkably limits its flexible use in practice.

The working principle of HA-mediated GISG depends on the oxidation-reduction reaction of HAuCl_4_ and NH_2_OH, i.e., 4HAuCl_4_ + 6NH_2_OH = 4Au(s) + 3N_2_O + 3H_2_O + 16HCl 44. Considering the different reactivities of NH_2_OH and NH_3_OH^+^ to Au^3+^ the reaction kinetics of Au^3+^ with HA is expected to be sensitive to the solution pH because NH_2_OH and NH_3_OH^+^ are present in its aqueous solution 45. From the kinetic viewpoint of redox reaction, the reduction efficiency of Au^3+^ to Au atoms by HA is remarkably reduced or even suppressed with decreased solution pH because the protonation of the amino group of HA results in increased NH_3_OH^+^ production with low reduction power. In addition, the protonation of HA at acidic pH can reduce the electron density between N and O atoms, resulting in difficult donation of electrons to Au^3+^ to promote its crystallization and further growth 46, 47. Therefore, the regulation of HA reducibility by solution pH provides an opportunity to control the nucleation and growth kinetics of Au^3+^. Under an appropriate acidic pH, the spontaneous nucleation of Au^3+^ by HA is completely prevented. By contrast, Au crystallization and *in situ* growth on the surface of AuNP probes can be achieved with Au surface-synergized catalytic reduction attributed to the decreased free energy of redox reaction, thus favoring the robustness and reproducibility of GISG-based signal amplification.

**Figure 1:**
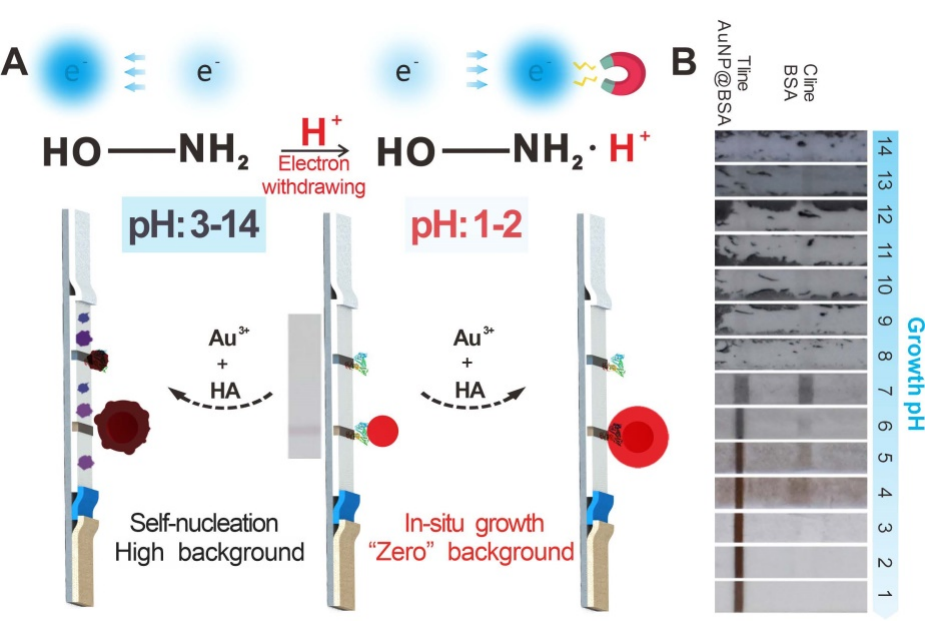
** (A)** Schematic of the developed pH-regulated gold *in situ* growth-mediated signal amplification of strip nanobiosensor without any background. (**B**) Images of immunochromatographic test strips for AuNP growth at different pH values.

Herein, we report an improved HA-mediated GISG signal amplification strategy to enhance the detection performance of ICA strip substantially. **Figure 1A** depicts the working mechanism of this GISG-amplified ICA method. In contrast to the traditional GISG method conducted at high pH (i.e., 7.0), our improved GISG technology is enabled by simply adjusting the solution pH to an acidic condition (i.e., 2.0) to suppress free reduction and self-nucleation of Au^3+^ and ensure gold crystallization and growth on the surface of AuNP probes with the aid of AuNP synergistic reduction (**Figure 1B**). This strategy can markedly enlarge the size of AuNPs accumulated on the T and control (C) lines by the antigen-antibody reaction to enhance colorimetric signal intensity with low and even “zero” background signals. The proposed GISG strategy is carried out at different pH values to explore the effect of solution pH on HA reducibility and Au^3+^ reaction kinetics. The nucleation and growth of Au^3+^ with and without AuNP probes are characterized by monitoring the consumption rate of Au^3+^ and the amount of gold crystallizations. Additionally, N-tert-butylhydroxylamine (N-HA) and N-hydroxyacetamide (A-HA) consisting of electron-donating (tertiary butyl) and electron-withdrawing (acetyl) groups, respectively, on the amino group, are selected as HA substitutes to investigate the reducing mechanism of HA by pH regulation. By combining with the ICA strip, the performance of the proposed pH-controlled GISG strategy in terms of sensitivity, background, selectivity, reproducibility, and practicality is characterized. Collectively, the improved GISG strategy provides unique superiorities over the conventional GISG method in ICA applications, including low background, robust operation, and good reproducibility and holds remarkable promise as a versatile signal amplification technology to drive the evolution and upgrade of AuNP-ICA.

## Results and Discussion

**Figure 2:**
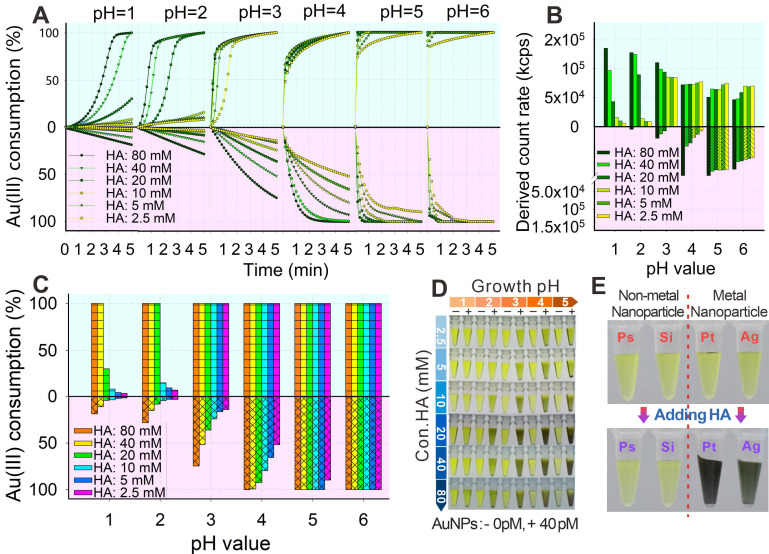
(**A**) Consumption kinetic curves of Au^3+^ in a series of concentrations of HA at different pH values within 5 min (thin cyan: with AuNP, lavender: without AuNP). (**B**) Consumption of Au^3+^ at 5 min under different pH values and concentrations of HA. (**C**) Intensity of scattered light of AuNP growth (with or without AuNPs) at 5 min under different pH values and concentrations of HA. (**D**) AuNP growth at 5 min under different pH values and concentrations of HA (+: with AuNPs, -: without AuNPs). (**E**) AuNP growth in different nanospheres before and after adding HA for 10 min.

The HA-mediated GISG method was performed by mixing 0.02% HAuCl_4_ with a series of HA concentrations under different pH conditions to confirm the pH-regulated reducibility. The consumption of Au^3+^ was used to monitor the process of gold crystallization and growth because the Au^3+^ concentration in the growth solution sharply decreased with the reduction of Au^3+^ into AuNPs. The Au^3+^ concentration was determined by measuring the characteristic absorption peak of HAuCl_4_ at 314 nm in accordance with a previous report (**Figure S1**) 48. Furthermore, the gold crystallization and growth were characterized by analyzing the light scattering intensity of growth solution via a dynamic light scattering (DLS) analyzer 49. The AuNPs produced from gold nucleation growth generated evident light scattering signals, whereas the gold growth solution containing chloric acid and HA does not. Results (**Figure 2A**, lavender area) showed that the consumption rate of Au^3+^ was related to solution pH, HA concentration, and growth time. When the solution pH was higher than 3.0, an evident light scattering signal in the gold growth solution was detected, indicating the occurrence of Au self-nucleation. This light scattering signal was remarkably enhanced with increasing solution pH and HA concentration (**Figure 2B**, lavender area). Furthermore, when the solution pH was raised to 6.0, Au^3+^ was totally consumed even at an extremely low HA concentration of 2.5 mM (**Figure 2C**), indicating the enhanced reducibility of HA with increasing solution pH. However, when the solution pH was decreased to 2.0, the Au^3+^ consumption was remarkably suppressed (**Figure 2A**), and almost no gold self-nucleation was observed even at long gold growth time (5 min) and high HA concentration (60 mM, **Figure 2C**). Moreover, we found that about 15%-25% Au^3+^ consumption was determined at pH 2.0 when HA concentration was as high as 80 mM, whereas almost no gold self-nucleation was observed (**Figure 2C**). It is supposed that Au^3+^ was reduced by HA into the transition nonionic state (Au^0^), thereby resulting in low absorbance at 314 nm 50, 51. A similar phenomenon was observed at pH 3.0 with HA concentration below 20 mM. In addition, we found that the consumption rates of Au^3+^ remarkably increased after the addition of AuNP probes (bovine serum albumin [BSA]-coated AuNPs, abbreviated as AuNP@BSA) in the gold growth solution (thin cyan area of **Figures 2A** and **2C**), indicating that the presence of AuNPs could promote the reduction of Au^3+^ into Au atoms and eventually form gold shell on the surface of AuNP probes. This finding was consistent with that of a previous report. It should be noticed thatthe suppressed crystallization and growth of Au^3+^ could be rebooted at solution pH of 2.0 and HA concentration of 40-60 mM after the addition of AuNP probes in the growth solution (**Figure 2C**), thus amplifying the AuNP signal with the low and even “zero” background on the test strip. The nucleation and growth of Au^3+^ were further explored by recording the color variances of growth solution at different pH values and HA concentrations. The concentration of HAuCl_4_ in this reaction system was increased to 30 mM to show the color difference. Results (**Figure 2D**) showed that at solution pH of 2.0, the color of growth solution without AuNP probes remained unchanged even at a high HA concentration (i.e., 80 mM). By contrast, after the addition of AuNP probes, the color of growth solution gradually changed to brownish red with increasing HA concentration, and evident brownish red precipitates were observed at the bottom of the test tube at HA concentration higher than 20 mM. However, when the solution pH increased to 4.0, an apparent color change was observed at HA concentration larger than 20 mM even without the addition of AuNP probes, suggesting the evident self-nucleation of Au^3+^ in this reaction system.

To evaluate the specificity of the synergic catalysis, we further mixed polystyrene (PsNPs), silica (SiO_2_NPs), platinum (PtNPs), and silver (AgNPs) nanoparticles with the gold growth solution containing 0.5% HAuCl_4_ and 40 mM HA at pH 2.0. Results (**Figure 2E**) showed that the gold growth solutions containing PtNPs and AgNPs were brownish black, whereas those containing PsNPs and SiO_2_NPs remained yellow. These phenomena showed that the AuNPs, PtNPs, and AgNPs could promote the nucleation growth of Au^3+^, whereas nonmetal nanoparticles could not facilitate the crystallization of Au^3+^, which indicated that only the presence of noble metal nanomaterials could synergistically promote the crystallization and growth of gold by reducing the free energy of redox reaction. The above results further verified that HA reducibility could be precisely controlled by simply adjusting the solution pH and that the self-nucleation of Au^3+^ triggered by HA was effectively suppressed at pH 2.0. Interestingly, the reducibility of HA to mediate the gold crystallization and growth could be regained with the aid of noble metal surface-synergized catalysis.

**Figure 3:**
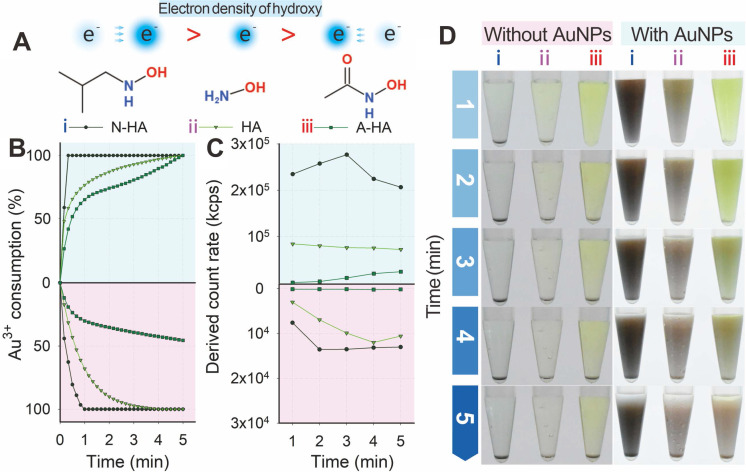
(**A**) Electron densities of hydroxy for N-HA, HA, and A-HA. (**B**) Consumption kinetic curves of Au^3+^. (**C**) Light intensity and (**D**) AuNP growth after adding HA (ⅱ) and N-HA (ⅰ) or A-HA (ⅲ) under conditions of pH 4 and 40 mM for 5 min.

Two structural analogs, namely, N-HA and A-HA (**Figure 3A**), were designed as HA alternatives to investigate the nucleation and growth of Au^3+^ and further clarify the mechanism of HA-mediated GISG method. N-HA and A-HA contained electron-donating (tertiary butyl) and electron-withdrawing (acetyl) groups, respectively, on the amino group. The introduction of a tert-butyl or acetyl group could change the electron density of hydroxyl groups and result in increased or decreased reducibility. The two analogs were used as reducing reagents to mediate the gold crystallization and growth at pH 4.0 and verify this hypothesis. For comparison, HA was used as control group. **Figure 3B** indicates that regardless of the presence of AuNP probes, the consumption of Au^3+^ was distinctly promoted by N-HA but suppressed by A-HA. These results were confirmed by determining the light scattering intensity of the gold growth solution (**Figure 3C**). **Figure 3D** shows that regardless of the presence of existing AuNP probes, the color intensities of growth solutions with different reducing agents followed the order: growth solution with N-HA as reducing reagent > growth solution with HA as reducing reagent > growth solution with A-HA as reducing reagent. After the addition of AuNP probes, the light scattering intensity of N-HA-mediated GISG method showed a significant decrease with prolonged reduction time to 3 min (**Figure 3B**). The possible reason was that the overgrowth of AuNPs with a large size was prone to precipitation due to its poor colloidal stability. These results demonstrated that the reducibility of HA was determined by the electron density of hydroxyl group, thus providing an opportunity to control the nucleation and growth of gold precisely.

**Figure 4:**
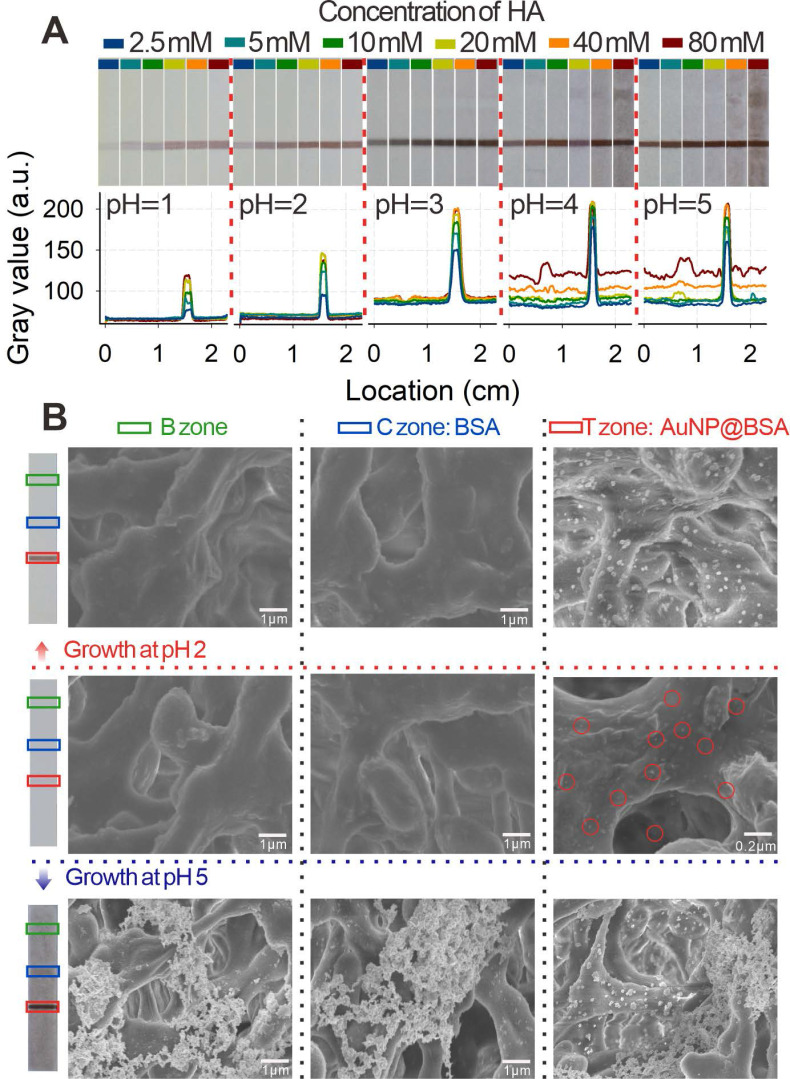
(**A**) Images and gray values of immunochromatographic test strips for AuNP growth by prespraying the C and T lines with the color of the small gold particle invisible under the conditions of different pH values and continuously reduced concentration of HA. (**B**) SEM images of 5 min growth after subjecting different pH values and 40 mM HA to the original pre-sprayed immunochromatographic test strips with small-diameter AuNP. Here, two typical pH values of 2 and 5 are selected.

Subsequently, the pH-regulated GISG strategy was further verified on the strip by spraying AuNP@BSA on the nitrocellulose (NC) membrane as T line. The GISG strategy was executed by immersing the NC membrane in a premixed growth solution containing 1% HAuCl_4_ and a series of HA concentrations (2.5, 5, 10, 20, 40, and 80 mM) with pH values at 1.0, 2.0, 3.0, 4.0, and 5.0. After incubation for 30 min, the membrane was scanned using a commercial colloidal gold strip reader. The optical density of the T line (OD_T_) and the background signal of the NC membrane were used to characterize the signal amplification and self-nucleation, respectively. As shown in **Figure 4A**, at each studied pH of the solution, the T line color and OD_T_ increased remarkably as the HA concentration rose, indicating that the high concentration of HA contributed to the crystallization and growth of gold. Moreover, we found that the generation of background signal at the NC membrane was closely related to the pH of growth solution. At solution pH of 1.0-2.0, the background color of the NC membrane had negligible change even at HA concentration as high as 80 mM compared with that of the untreated NC membrane (**Figure 4A**), and this finding was further confirmed by the low and constant noise signal recorded by the strip reader. However, by increasing the solution pH to 3.0, the background color of NC membrane gradually changed into weak brown-red with increased noise signal. Additionally, the background color and noise continued to increase with further increase in solution pH especially with increasing HA concentration. For example, at solution pH of 4.0 and HA concentration of 40 mM, the color of the NC membrane further deepened with many randomly distributed brown-red stains (**Figure 4A**), thus resulting in evident background color and signal interference for reducing the signal-to-noise ratio and producing false-positive results. These observations further proved that the self-nucleation of Au^3+^ on the test strip could be effectively suppressed by lowering the pH of the growth solution below 2.0, which was attributed to the intrinsic weak reducibility of HA at low pH. This result was consistent with that obtained by the test tube experiment.

The nucleation and growth of Au^3+^ on the NC membrane was further characterized by observing the T, C, and blank (B) zones of the strip via scanning electron microscopy (SEM) after conducting the GISG process at pH 2.0 and 5.0. T and C lines were sprayed with 20 pmol L^-1^ AuNP@BSA and 1 mg mL^-1^ BSA solution, respectively. **Figure 4B** showed that the strip without executing the GISG strategy only exhibited small-sized AuNPs evenly dispersed on the T zone (marked with red ring). Moreover, no AuNP was observed at B and C zones. After performing the GISG method at pH 2.0 and 5.0, a mass of AuNPs was found to aggregate on the T line, and the size of AuNPs was significantly enlarged from 20 nm to 100 nm. These phenomena suggested that the GISG-based signal amplification was derived from the enlargement of AuNP size. Notably, no AuNP was observed at the B and C zones after executing the GISG strategy at pH 2.0, whereas numerous AuNPs were found on these areas at pH 5. These findings further verified that the low pH of growth solution for the GISG (e.g., 2.0) could totally suppress the self-nucleation of Au^3+^ on the strip, thus ensuring low background, good robustness, and high reproducibility.

**Figure 5:**
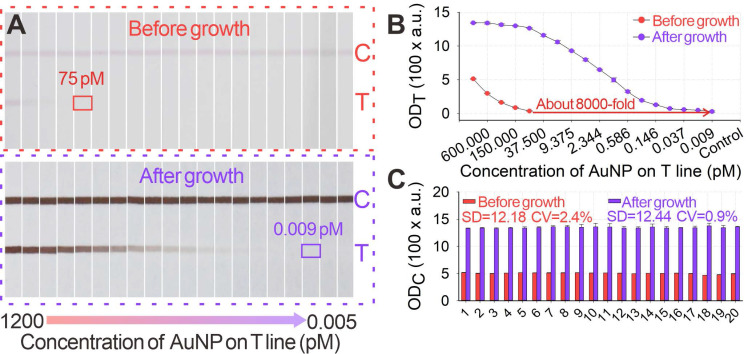
(**A**) Images of original small-diameter AuNP and AuNP growth on strips for 12 min under the conditions of pH 2, 40 mM HA, and 0.5% HAuCl_4_. (**B**) Amplification curves of OD_T_ value after AuNP growth. (**C**) OD_C_ values before and after AuNP growth.

Given its unique advantages over the traditional GISG technique, the signal amplification performance of our proposed pH-regulated GISG method was further evaluated. Several key parameters that affected the signal amplification intensity, including the concentrations of HAuCl_4_ and HA and the reduction reaction time, were optimized at growth solution pH of 2.0. The difference in OD_T_ (ΔOD_T_) before and after the GISG treatment was used to achieve optimum growth conditions. The results in **Figures S2A-C** indicated that the optimum combinations were as follows: HAuCl_4_ and HA concentrations of 0.5% and 40 mM, respectively, and reaction time of 10 min, thus enabling maximal signals on the T line. The robustness of this GISG strategy was evaluated by soaking the strip in the growth solution for 2 h, and results in **Figures S3A-B** showed negligible signal changes in the OD values on the B and C areas (sprayed with BSA solution). The signal amplification potential of our developed GISG method was determined by spraying a series of AuNP@BSA within the concentration range of 0-1200 pM on the strip as T line and with 1200 pM AuNP@BSA as C line. **Figures 5A-B** displayed that with or without GISG treatment, the T line color and OD_T_ of the strip gradually reduced as the AuNP@BSA concentration decreased, indicating that the GISG process did not affect the concentration-response relationship. However, the lowest response concentration of AuNP@BSA after growth was significantly decreased to 0.009 pM, which showed approximately 8333-fold improvement compared with that of the unamplified strip (75 pM). This result displayed ultrahigh signal amplification capability of the designed GISG method. The reproducibility of this pH-regulated GISG method was monitored by recording the OD values on the C line (OD_C_) of 20 test strips before and after growth. **Figure 5C** shows a remarkable increase in OD_C_ from 507.5 ± 12.18 to 1382.2 ± 12.44 after GISG amplification with a variance coefficient (CV) of 0.9%. This low CV was comparable to that obtained before amplification (2.4%), implying the good reproducibility of our reported GISG strategy. The universality of the developed GISG method was characterized by spraying other types of signal labels, including PsNPs@BSA, SiO_2_NPs@BSA, PtNPs@BSA, and AgNPs@BSA, on the NC membrane as T lines. **Figure S4** reveals that only the test strips immobilized with PtNPs@BSA and AgNPs@BSA produced significant signal increments on the T line, indicating excellent selectivity of the proposed GISG strategy for enhancing the signal and detection sensitivity of noble metal nanoparticle-based test strips. These results confirmed that the pH-regulated GISG strategy had many advantages in terms of signal amplification, reproducibility, universality, and selectivity and showed remarkable potential for enhancing the sensitivity of conventional AuNP-ICA.

**Figure 6:**
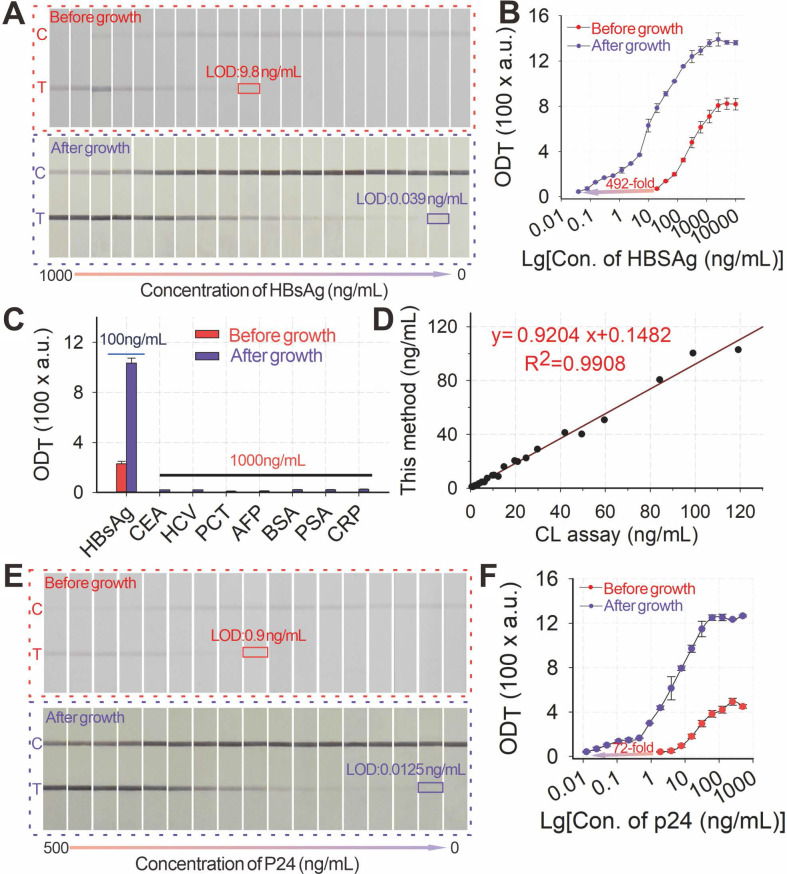
(**A**) Images of immunochromatographic strips for HBsAg detection before and after growth under the conditions of pH 2, 40 mM HA, and 0.5% HAuCl_4_. (**B**) Detection curves for HBsAg detection before and after growth. (**C**) Selectivity evaluation of the proposed method by determining signal responses against several common protein biomarkers in serum, including HBsAg (100 ng mL^-1^) and other nontargets, i.e., CEA, HCV, PCT, AFP, BSA, PSA, and CRP, at 1 μg mL^-1^. (**D**)Correlation analysis of the measured HBsAg concentrations between this proposed method and the clinically used HBsAg chemiluminescence assay kits in 28 human serum samples with target concentrations ranging from 0 ng mL^-1^ to 119.2 ng mL^-1^. (**E**) Images of immunochromatographic strips for P24 detection before and after growth under the conditions of pH 2, 40 mM HA, and 0.5% HAuCl_4_. (**F**) Detection curves for P24 detection before and after growth.

Given its high signal amplification ability, the pH-regulated GISG method was further configured into the AuNP-ICA platform to improve the detection of hepatitis B surface antigen (HBsAg), the well-accepted serologic marker for the POC diagnosis of hepatitis B virus infection 52. The development and optimization of AuNP-ICA included pH (**Figure S5A**) and amounts of anti-HBsAg mAbs for the preparation of AuNP probes (**Figure S5B**). The quantitative detection of HBsAg by using AuNP-ICA was performed by running a series of HBsAg solutions with concentrations ranging from 0 ng mL^-1^ to 5000 ng mL^-1^. **Figures 6A-B** show that the LOD of unamplified AuNP-ICA for HBsAg detection was 9.75 ng mL^-1^. After growth amplification, the LOD was down to 0.0198 ng mL^-1^, which was a 500-fold enhancement in sensitivity compared with that of unamplified AuNP-ICA. The amplified AuNP-ICA exhibited a good dynamic detection range from 0.039 ng mL^-1^ to 625 ng mL^-1^. The selectivity of AuNP-ICA was evaluated by determining six other serum biomarkers (1000 ng mL^-1^), namely, carcinoembryonic antigen, hepatitis C surface antigen, procalcitonin (PCT), alpha fetoprotein (AFP), prostate-specific antigen (PSA), and C-reactive protein (CRP). **Figure 6C** shows that the strips before and after growth amplification could selectively respond to HBsAg even at a 10-fold lower concentration over other serum biomarkers, confirming high selectivity of the proposed GISG method for HBsAg without any false-positive result. The precision and accuracy of the proposed amplified AuNP-ICA were estimated by determining the recoveries and CV of five spiked serum samples. As shown in **Table S1**, the average recoveries ranged from 82% to 120%, and CV values were 0.7% to 13.2%, indicating the acceptability of using the proposed GISG amplification strategy for the accurate and sensitive quantitative detection of HBsAg. The reliability of this amplified approach was further validated through a commercial chemiluminescence immunoassay kit by simultaneously analyzing 28 actual clinical serum samples (**Figure 6D**).

The pH-regulated GISG method was further extended to enhance the POC testing of the HIV-1 capsid p24 antigen, the earliest biomarker after acute HIV infection to verify its universality. The early and sensitive detection of the p24 antigen could largely shorten the diagnostic window of HIV infection to promote timely intervention and interrupt virus transmission 53. The detection of the p24 antigen by integrating the pH-regulated GISG amplification with the AuNP-ICA was evaluated (**Figures 6E-F**), thereby resulting in LOD of 12.5 pg mL^-1^. This LOD was about 72-fold enhancement in sensitivity the unamplified AuNP-ICA (900 pg mL^-1^) under the developed conditions (**Figure S6A-B**). Notably, the whole assay time for running the strip and performing the GISG procedure was only 20 min, which was still suitable for POC applications. These results corroborated the feasibility for the real-world applications of the designed pH-regulated GISG strategy.

## Conclusion

We successfully demonstrate the use of pH-regulated GISG strategy as a controlled signal amplification technology for *in vitro* diagnostic assays by suppressing the gold self-nucleation to achieve the low and even “zero” background, sensitivity, and reproducibility. We further clarify the mechanism of pH to regulate the gold growth by affecting the electron density of the hydroxyl group to vary HA reducibility. Given its advantages of high sensitivity, “zero” background, robust operation, and good reproducibility, this system is configured into a sandwich AuNP-ICA format to achieve the ultrasensitive immunochromatographic diagnostics of viral infections. The LOD values of the designed GISG-amplified AuNP-ICA are as low as 0.0198 ng mL^-1^ for HBsAg and 0.0125 ng mL^-1^ for p24, which are lower by about 500- and 70-fold, respectively, than those of the unamplified AuNP-ICA. Moreover, these LOD values are comparable with those of routinely used laboratory diagnostic techniques, but the speed and portability are not compromised. The incorporation of pH-regulated GISG technology into paper-based lateral flow microfluidics can provide a sensitive and robust POC tool for the *in vitro* diagnostics of diseases and has the potential to redefine immunochromatographic diagnostics.

## Supplementary Material

Supplementary methods, figures, and table.
