# Supplementary material for: Avoiding the self-nucleation interference: a pH-regulated gold in situ growth strategy to enable ultrasensitive immunochromatographic diagnostics
Source: Theranostics. 2022 Mar 14;12(6):2801–10. doi: 10.7150/thno.70092 (PMC8965500; doi:10.7150/thno.70092)
Supplement: Supplementary file 1 — Supplementary methods, figures, and table. [file thnov12p2801s1.pdf]

Supporting Information for:

Avoiding the self-nucleation interference: a pH-regulated gold in situ growth strategy to enable ultrasensitive immunochromatographic diagnostics

Hong Duan[a‡], Tongtong Ma[a‡], Xiaolin Huang*[a], Bao Gao[a], Lingyan Zheng[b], Xirui Chen[a], Yonghua Xiong*[a], Xiaoyuan Chen[c, d, e]

[a] State Key Laboratory of Food Science and Technology, School of Food Science and Technology, Nanchang University, Nanchang 330047, P. R. China

[b] Beijing Engineering and Technology Research Center of Food Additives, Beijing Technology & Business University, Beijing 100048, P. R. China

[c] Departments of Diagnostic Radiology, Surgery, Chemical and Biomolecular Engineering, and Biomedical Engineering, Yong Loo Lin School of Medicine and Faculty of Engineering, National University of Singapore, Singapore, 119074, Singapore

[d] Clinical Imaging Research Centre, Centre for Translational Medicine, Yong Loo Lin School of Medicine, National University of Singapore, Singapore 117599, Singapore

[e] Nanomedicine Translational Research Program, NUS Center for Nanomedicine, Yong Loo Lin School of Medicine, National University of Singapore, Singapore 117597, Singapore

[‡]These authors contributed equally to this work.

*Corresponding authors: Dr. Xiaolin Huang and Dr. Yonghua Xiong

E-mail: hxl19880503@163.com (X. H.); yhxiongchen@163.com (Y. X.)

1. Experimental procedures

S1 Materials

Hydrogen tetrachloroaurate (III) trihydrate ($HAuCl_4$•$3H_2O$, 99.9%) and trisodium citrate ($Na_3C_6H_5O_7$•$2H_2O$) were purchased from Sigma-Aldrich (St. Louis, MO, USA) to synthesize AuNP. Bovine serum albumin (BSA), hydroxylamine hydrochloride (NH_2OH•HCl), procalcitonin (PCT), prostate-specific antigen (PSA), Creactive protein (CRP), and alpha-fetoprotein (AFP) were purchased from Sigma-Aldrich (St. Louis, MO, USA). sample pad, nitrocellulose (NC) membrane, and absorbent pad were obtained from Schleicher and Schuell GmbH (Dassel, Germany). Goat anti-mouse IgG antibodies were purchased from Chongqing Xinyuanjiahe Biotechnology, Inc. (Chongqing, China). Anti-p24-captured polyclonal antibodies (anti-p24 cAbs, 5.0 mg mL^{-1}) and anti-p24-detected monoclonal antibodies (anti-p24 dAbs, 6.4 mg mL^{-1}) were provided by Abcam (Abcam, Cambridge, MA). Positive and negative HBsAg serum samples were collected from the People's Hospital of Nanchang. The purified Milli-Q water prepared from the Milli-Q system was used throughout this study (Millipore, Milford, MA, USA). Phosphate buffer (PB, 0.1 M) was prepared by adding 35.8 g $Na_2HPO_4 \cdot 12H_2O$ and 15.6 g $NaH_2PO_4 \cdot 2H_2O$ in 1000 mL Milli-Q water. pH was adjusted to 7.4 before use unless otherwise specified. All other analytical-grade chemicals were bought from Sinopharm Chemical Corp. (Shanghai, China) and used without further purification.

S2 Instrumentation

UV–visible absorption spectra were obtained on a UV–vis spectrophotometer (Thermo Fisher, G10S, USA). DLS analysis was conducted using the Malvern Nano-Z90 zetasizer (Malvern Instruments, Worcestershire, UK). The morphological characteristics and size of the nanoparticles were determined using the JEOL (JEM 2100, **A**kishima, **T**okyo) transmission electron microscope and Hitachi SEM (S-4800). The commercial HG-8 strip reader was obtained from Shanghai Huguo Science Instrument Co., Ltd. (Shanghai, China). The BioDot XYZ platform combined with a motion controller, the BioJet Quanti3000k dispenser, and the AirJet Quanti3000k

dispenser for solution dispensing were supplied by BioDot (Irvine, CA). An automatic programmable cutter was purchased from Jinbiao Biotechnology Co., Ltd. (Shanghai, China).

S3 Synthesis of 18 nm citrate-coated AuNPs

Citrate-coated AuNPs were synthesized using the citrate reduction method described in our previous work.[1] In a typical synthesis, 2.7 mL of 1% sodium citrate solution was added to 100 mL of boiling 0.01% gold chloride trihydrate solution under constant stirring. Citrate-coated AuNPs (18 nm) were obtained after 10 min of reaction when the color of the solution changed from light yellow to red purple.

S4 Preparation of anti-p24 dAbs@AuNP probes

The probes of p24 were prepared by immobilizing anti-p24 dAbs on the surface of citrate-coated AuNP via electrostatic adsorption.[2] Typically, the pH of 1 mL citrate-coated AuNP solution was adjusted to 8.5 with 0.01 M K_2CO_3, added dropwise with 5 μg anti-p24 dAbs solution after 1 h incubation under gentle stirring at 25 °C, and added with 500 μL of 10% BSA (*w/v*). The mixed solution was incubated for another 60 min. The as-prepared anti-p24 dAbs@AuNP was then purified via centrifugation and resuspended in 0.01 M PB buffer (pH = 7.4) containing 25% sucrose and 0.1% sodium azide and stored at 4 °C until further use.

S5 Preparation of anti-HBsAg dAbs@AuNP probes

The probes of HBsAg were prepared by immobilizing anti-HBsAg dAbs on the surface of the citrate-coated AuNP via electrostatic adsorption.[2] Typically, the pH of 1 mL citrate-coated AuNP solution was adjusted to 6.0 with 0.01 M K_2CO_3, added dropwise with 5 μg anti-HBsAg dAbs solution after 1 h incubation under gentle stirring at 25 °C, and added with 500 μL of 10% (*w/v*) BSA. The mixed solution was incubated for another 60 min. The as-prepared anti-HBsAg dAbs@AuNP was then purified via centrifugation and resuspended in 0.01 M PB buffer (pH = 7.4) containing 25% sucrose and 0.1% sodium azide and stored at 4 °C until further use.

S6 Fabrication of GISG-amplified AuNP based test strip

Similar to traditional AuNP-based test strip, the proposed GISG-amplified AuNP

based test strip were composed of the following parts: a sample pad, NC membrane, and absorbent pad. Anti-HBsAg/p24 cAbs (1.0 mg mL^{-1}) and goat antimouse IgG (1.0 mg mL^{-1}) prediluted in 0.01 M PB buffer (pH = 6.0) were spotted onto NC membranes as a test (T) line and a control (C) line at a distance of 4 mm. The sample pad, NC membrane, and absorption pad were then assembled into a plastic backing plate, cut into 4 mm wide strips, and packaged in a plastic casing for subsequent storage in a drying cylinder at room temperature.

S7 HBsAg detection through GISG-amplified AuNP-ICA

In this experiment, 2 μL as-prepared detection probes (anti-HBsAg dAbs@AuNP, 5.85 nM) were added to 70 μL of 0.01 M PB buffer solution (pH 7.4) containing target HBsAg concentrations of 5000, 2500, 1250, 625, 312.5, 156.25, 78.15, 39, 19.5, 9.75, 4.9, 2.45, 1.2, 0.6, 0.3, 0.15, 0.078, 0.039, 0.0198, and 0 ng mL$^{-□}$. The resulting mixture was incubated at room temperature for 5 min and added to the sample well of strip nanobiosensors for a 10 min reaction. Approximately 70 μL of 80 mM HA (pH = 2) and 0.5 wt.% $HAuCl_4 \cdot 3H_2O$ solution with equal volume were added to the sample well for another 10 min of reaction for the controlled growth of AuNP, thereby obtaining the effect of signal amplification further. The optical densities of the T (OD_T) and C (OD_C) lines in each cycle of the AuNP assembly were recorded using the commercial HG-8 strip reader, and the corresponding photograph of the reacted strips was obtained using a digital camera (Sony DX3400, Tokyo, Japan).

S8 p24 detection through GISG-amplified AuNP-ICA

In this experiment, 2 μL as-prepared detection probes (anti-p24 dAbs@AuNP, 5.85 nM) were added to 70 μL of 0.01 M PB buffer solution (pH 7.4) containing target p24 concentrations of 500, 250, 125, 62.5, 31.3, 15.6, 7.8, 3.9, 1.875, 0.9, 0.45, 0.22, 0.11, 0.05, 0.025, 0.0125, and 0 ng mL^{-1}. The resulting mixture was incubated at room temperature for 5 min and then added to the sample well of strip for a 10 min reaction. The controlled growth of HA methods was similar to HBsAg detection.

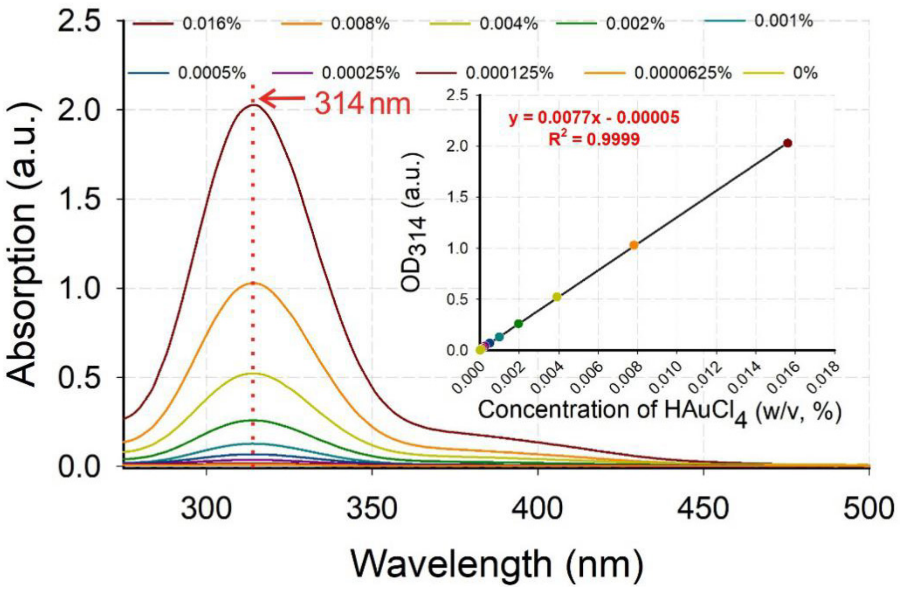

Figure S1. UV absorption spectra of Au^{3+} complex ions in different concentrations. (inset) Linear relationship between the absorbance at 314 nm of Au^{3+} complex ion and its concentration.

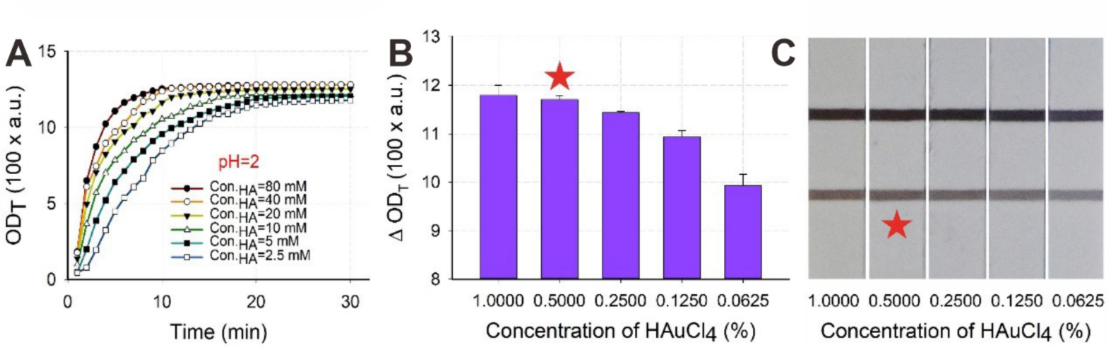

Figure S2. (A) Reaction kinetics of continuously reduced concentrations of HA within 30 min at pH 2. (B-C) Optimization of the amount of $HAuCl_4$ on immunochromatographic test strips under the conditions of pH 2 and 40 mM HA.

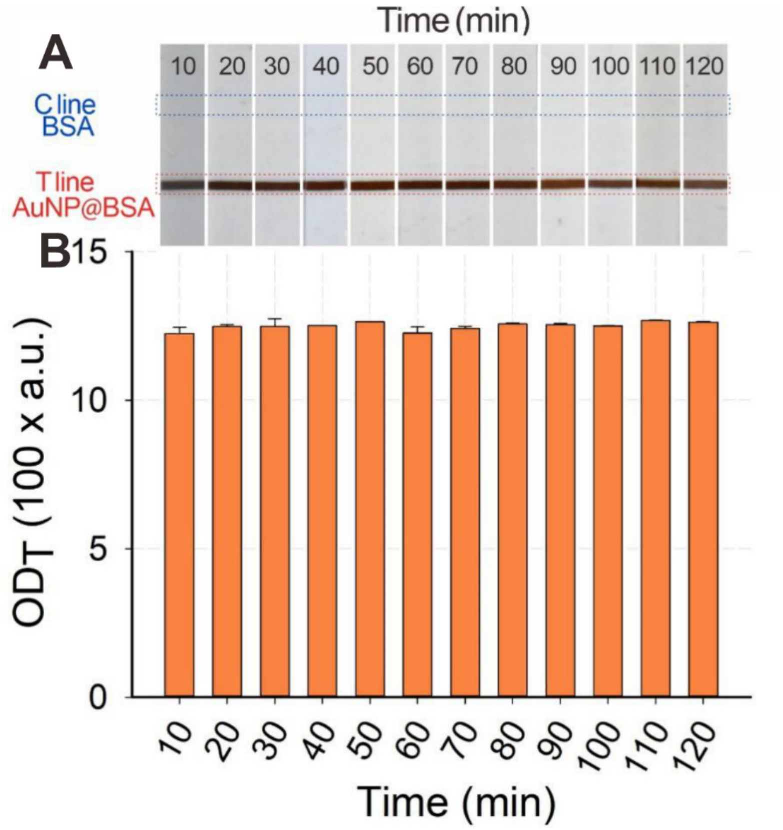

Figure S3. (A) Image and (B) OD_T value of the monitored AuNP growth for 2h. Pre-sprayed invisible small-diameter AuNP color T-line recorded every 10 minutes under the condition of pH=2, 40 mM HA.

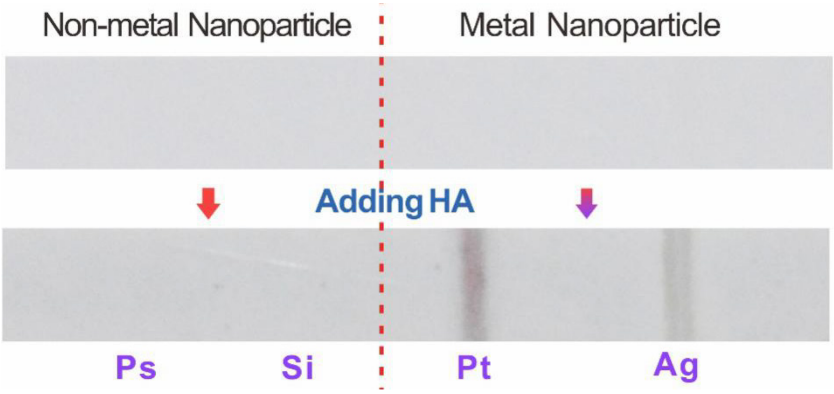

Figure S4. AuNP growth on immunochromatographic test strips with different nanospheres pre-sprayed with invisible color concentrations.

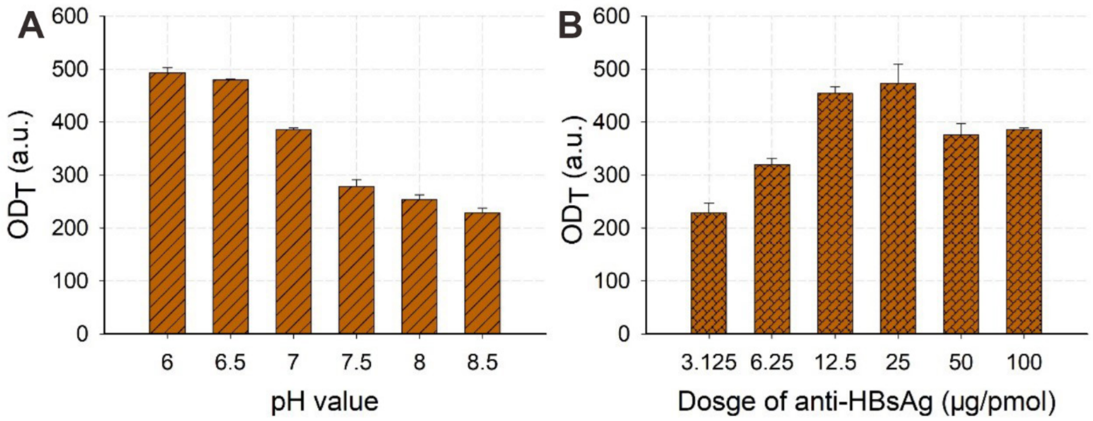

Figure S5. Parameter optimization for the preparation of AuNP@anti-HBsAg. OD_T value of test strips based on AuNP@anti-HBsAg prepared using different (A) pH values, which were adjusted by adding 0.01 M K_2CO_3 to the AuNP solution (100 pM), and (B) dosages of anti-HBsAg.

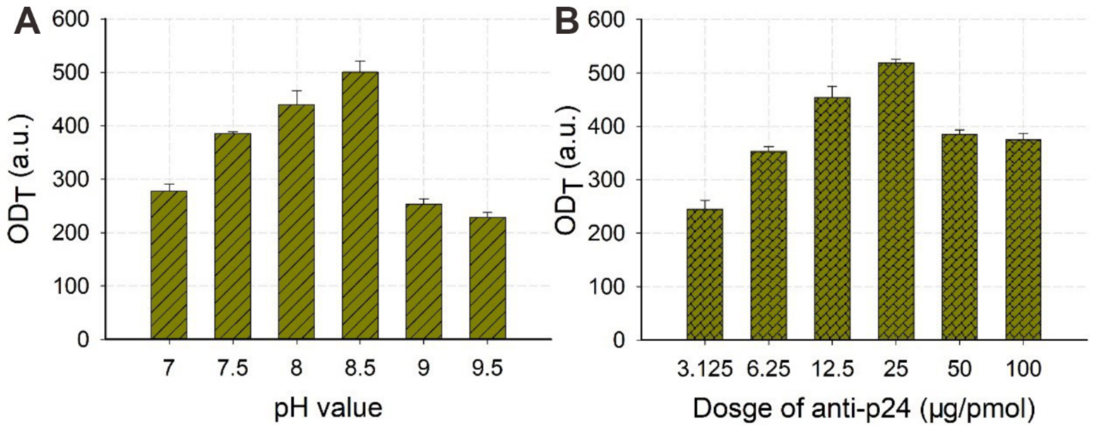

Figure S6. Parameter optimization for the preparation of AuNP@anti-p24. OD_T values of test strips based on AuNP@anti-p24 prepared using different (A) pH values, which were adjusted by adding 0.01 M K_2CO_3 to the AuNP solution (100 pM), and (B) dosages of anti-p24.

Table S1. Accuracy of pH-regulated amplification ICA for HBsAg detection.

HBsAg added (ng mL^{-1})	Intra-assay precision			Inter-assay precision[a]		
	Mean[b]	Recovery (%)	CV (%)	Mean[b]	Recovery (%)	CV (%)
500	468.6	93.5	5.8	511.5	102.1	9.9
50	45.9	92	7.3	48.5	97.7	7.4
5	5.6	112	2.9	5.8	114	0.7
0.5	0.43	86	9.3	0.6	120	12.5
0.05	0.04	82	11.3	0.04	82	13.2

[a] Assay was completed every day for three days.

[b] Mean value of four replicates at each diluted concentration.

References

1. Huang X, Zhou Y, Ding L, Yu G, Leng Y, Lai W, et al. Supramolecular Recognition-Mediated Layer-by-Layer Self-Assembled Gold Nanoparticles for Customized Sensitivity in Paper-Based Strip Nanobiosensors. Small. 2019; 15: 1903861.
2. Fu J, Zhou Y, Huang X, Zhang W, Wu Y, Fang H, et al. Dramatically Enhanced Immunochromatographic Assay Using Cascade Signal Amplification for Ultrasensitive Detection of *Escherichia coli* O157:H7 in Milk. J Agric Food Chem. 2020; 68: 1118-25.
